# A hybrid neural network approach for classifying diabetic retinopathy subtypes

**DOI:** 10.3389/fmed.2023.1293019

**Published:** 2024-01-04

**Authors:** Huanqing Xu, Xian Shao, Dandan Fang, Fangliang Huang

**Affiliations:** ^1^The School of Medical Information Engineering, Anhui University of Chinese Medicine, Hefei, China; ^2^NHC Key Laboratory of Hormones and Development, Chu Hsien-I Memorial Hospital and Tianjin Institute of Endocrinology, Tianjin Medical University, Tianjin, China; ^3^The School of Medical Information Engineering, Guangzhou University of Chinese Medicine, Guangzhou, China

**Keywords:** diabetic retinopathy classifications, hybrid neural network, EfficientNet, Swin Transformer, CAD systems

## Abstract

**Objective:**

Diabetic retinopathy is a prevalent complication among diabetic patients that, if not predicted and treated promptly, can lead to blindness. This paper proposes a method for accurately and swiftly predicting the degree of diabetic retinopathy using a hybrid neural network model. Timely prediction of diabetic retinopathy is crucial in preventing blindness associated with this condition.

**Methods:**

This study aims to enhance the prediction accuracy of diabetic retinopathy by utilizing the hybrid neural network model EfficientNet and Swin Transformer. The specific methodology includes: (1) combining local and global features to accurately capture lesion characteristics by leveraging the strengths of both Swin Transformer and EfficientNet models; (2) improving prediction accuracy through a comprehensive analysis of the model’s training details and applying data augmentation techniques such as Gaussian blur to enhance the hybrid model’s performance; (3) validating the effectiveness and utility of the proposed hybrid model for diabetic retinopathy detection through extensive experimental evaluations and comparisons with other deep learning models.

**Results:**

The hybrid model was trained and tested on the large-scale real-world diabetic retinopathy detection dataset APTOS 2019 Blindness Detection. The experimental results show that the hybrid model in this paper achieves the best results in all metrics, including sensitivity of 0.95, specificity of 0.98, accuracy of 0.97, and AUC of 0.97. The performance of the model is significantly improved compared to the mainstream methods currently employed. In addition, the model provides interpretable neural network details through class activation maps, which enables the visualization of diabetic retinopathy. This feature helps physicians to make more accurate diagnosis and treatment decisions. The model proposed in this paper shows higher accuracy in detecting and diagnosing diabetic retinopathy, which is crucial for the treatment and rehabilitation of diabetic patients.

**Conclusion:**

The hybrid neural network model based on EfficientNet and Swin Transformer significantly contributes to the prediction of diabetic retinopathy. By combining local and global features, the model achieves improved prediction accuracy. The validity and utility of the model are verified through experimental evaluations. This research provides robust support for the early diagnosis and treatment of diabetic patients.

## 1 Introduction

Diabetic retinopathy (DR) is one of the most common complications of diabetes and can lead to blindness if not predicted and treated in time. Therefore, rapid and accurate prediction of the degree of diabetic retinopathy is of great significance for preventing blindness. In the past few years, deep learning has become an essential tool for predicting DR with much success. In recent years, convolutional neural network (CNN) based models have been widely used for automatic diagnosis and prediction of DR. These models can learn and extract features in fundus images and use them for automatic classification and prediction of DR.

In an article published in the journal AMIA in 2016, Lam et al. (1) used a deep learning model to automatically detect DR, trained on a training set of multiple fundus images, and achieved an accuracy rate of 75.5% in the test set %. Keel et al. (2) used a new deep learning model capable of detecting DR lesions requiring further examination, trained on a training set of multiple fundus images, with an accuracy of 96% in the test set. He et al. (3) proposed a deep residual network (ResNet) model for image recognition and classification, which is also widely used in automatic diagnosis of DR. Shah et al. (4) used a CNN model capable of identifying diabetic patients with vision-threatening DR, trained on a training set of multiple fundus images, with an accuracy of 98.98% in the test set. Abràmoff et al. (5) used a deep learning model that could automatically detect the extent of DR and achieved high accuracy on public datasets. These studies show that the CNN model has high application value in DR prediction and can be effectively used for automatic diagnosis and prediction of DR. Applying these models can help improve the efficiency of early screening for DR, promote early intervention and treatment of DR, and thus help reduce vision loss and blindness caused by DR.

Abràmoff et al. (5) used a deep learning model that automatically detected the degree of DR and achieved high accuracy on public datasets. This study demonstrates that the CNN model can be used to automatically detect the extent of DR, thus helping to improve the diagnosis of DR. In recent years, attention-based models have been widely used for automatic diagnosis and prediction of DR. These models can adaptively focus on essential features in fundus images and use them for automatic classification and prediction of DR. Cao et al. (6) proposed a domain adaptation model that can efficiently and adaptively focus on essential features in fundus images and achieved high accuracy on public datasets. Du et al. (7) proposed a model based on the attention mechanism, which can simultaneously pay attention to local and global information in fundus images and achieve high accuracy in the test set. Tan et al. (8) proposed a spatial attention mechanism-based model that can adaptively focus on spatial features in fundus images and achieve high accuracy on multiple datasets. Zhang et al. (9) proposed a deep learning model based on an attention mechanism, which can adaptively focus on essential features in fundus images and achieve high accuracy in the test set. Yu et al. (10) proposed a deep residual network model based on an attention mechanism, which can adaptively focus on essential features in fundus images and achieve high accuracy on multiple datasets.

These studies demonstrate that attention-based models have high application value in DR prediction, which can adaptively focus on essential features in fundus images and improve the accuracy of automatic classification and prediction of DR. The application of these models can help improve the efficiency of early screening of DR and promote early intervention and treatment of DR, thereby helping to reduce vision loss caused by DR and reduce the waste and cost of medical resources. However, existing deep learning models still have some challenges processing fundus images. On the one hand, some convolutional neural network (CNN)-based models may not be able to capture global features in fundus images, resulting in inaccurate prediction results; on the other hand, some models based on attention mechanisms can capture global features, but for fundus images Local features in. Because the lesion is a diagnosis that must be combined with long-range and local pixel information, the diagnosis of diabetic retinopathy by expert doctors often requires local and global image features. Therefore, researchers need to develop a new deep-learning model that can process the local features of fundus images and capture the global features to improve the accuracy and reliability of DR prediction.

Based on these backgrounds and motivations, this paper proposes a diabetic retinopathy prediction method based on a hybrid neural network model, combining EfficientNet and Swin Transformer to better handle fundus images’ global and local features. The data enhancement algorithm is applied to the internal image to enhance the robustness of the model and enable faster identification of the lesion. By combining the two hybrid neural network models and data augmentation algorithms proposed in this paper, the prediction accuracy of DR is improved, providing doctors with more accurate diagnosis and treatment guidance, thus contributing to the prevention of blindness.

The prediction of diabetic retinopathy based on the hybrid neural network model EfficientNet and Swin Transformer has made essential contributions in the following three aspects:

1. Combining local and global features: since a lesion needs to be diagnosed by combining long-range pixel information, the model proposed in this paper uses two models, Swin Transformer and EfficientNet. The former can effectively extract global features, and the latter can be better. At the same time, the data enhancement algorithm is applied to the original eye image so that the lesion can be identified more quickly. By combining two hybrid models and data augmentation algorithms, this paper can more accurately capture the local and global features of diabetic retinopathy images and improve the recognition accuracy of the model.
2. Improve prediction accuracy: the combination of hybrid models improves the model’s prediction accuracy, which is better than a single model. The two models complement each other in terms of depth and computational efficiency. Through in-depth analysis of the training details of the model, some data enhancement methods are proposed to increase the robustness of the model, thereby improving the model’s performance.
3. A comprehensive experimental evaluation of publicly available datasets and comparisons with other advanced deep learning models demonstrate the effectiveness and practicality of the hybrid model proposed in this paper for the detection of diabetic retinopathy.

## 2 Literature review

### 2.1 Overview of diabetic retinopathy and its detection

Diabetic retinopathy is a common complication of diabetes that can lead to vision loss and even blindness. Early detection and treatment of diabetic retinopathy is critical to preventing permanent vision loss. In recent years, more and more studies have focused on using computer-aided diagnosis (CAD) systems to aid in detecting and diagnosing diabetic retinopathy.

This literature review provides an overview of diabetic retinopathy and its detection, focusing on recent advances in CAD systems. Many studies have used deep learning techniques such as convolutional neural networks (CNN) to detect diabetic retinopathy from fundus images and obtained encouraging results. These CNN-based systems (1–5) have been shown to have high sensitivity and specificity, some even outperforming human experts. In addition to the CNN method, other machine-learning techniques have also been used to detect diabetic retinopathy, including support vector machines (SVM), decision trees, and random forests (11). Some studies have also explored image processing techniques to extract features from fundus images, which are input to machine learning models. Despite the promising performance of CAD systems for the detection of diabetic retinopathy, there are still some challenges that need to be addressed. A significant challenge is the lack of large-scale and diverse datasets to train and validate these systems. Another challenge is the generalization performance of these systems across different populations and imaging modalities. In conclusion, CAD systems based on deep learning and other machine learning techniques show great potential in detecting and diagnosing diabetic retinopathy. However, further research is still needed to address unresolved challenges and optimize the performance of these systems for clinical applications.

### 2.2 Review of deep learning models for diabetic retinopathy detection

In recent years, with the development of deep learning, deep learning-based diabetic retinopathy (DR) detection models have received more and more attention. This paper reviews related research literature and introduces their main features and contributions.

First, some studies have implemented automatic detection of DR using a convolutional neural network (CNN). Some models use transfer learning technology to apply pre-trained models to DR detection and achieve good results. For example, the model proposed by Bhardwaj et al. (12) achieved a detection accuracy of 90.51% when processing images. However, these models may ignore some detailed information because traditional convolutional neural networks mainly focus on local features, and it is difficult to notice the long-range image information of images. Second, some studies introduce attention mechanisms (5–7) into DR detection models. These models can adaptively focus on important image parts and achieve better performance. Some researchers (8–10) employ attention mechanisms to select the most representative image regions to improve the accuracy of DR detection. Deep learning models have broad application prospects in DR detection.

### 2.3 Overview of EfficientNet and Swin Transformer

EfficientNet and Swin Transformer are two models that have received much attention in deep learning in recent years. EfficientNet (13) is an efficient convolutional neural network model the Google research team proposed in 2019. It achieves higher model efficiency by combining different network depths, widths, and resolutions. Swin Transformer is a new type of Transformer model proposed, which improves model performance by introducing a local window attention mechanism and cross-stage information exchange. This article will review the development history and characteristics of the EfficientNet and Swin Transformer models and their application to image classification tasks. The article (14) proposes a new visual Transformer network architecture, Swin Transformer, which introduces a translation window mechanism and achieves efficient image feature extraction through a hierarchical attention mechanism. Swin Transformer has achieved good results on multiple computer vision tasks and performs better than other traditional CNN and Transformer models. The emergence of EfficientNet has solved the contradiction between the model size and the amount of calculation in the deep neural network to a certain extent. It uses compound scaling to balance model complexity and performance by adjusting network depth, width, and resolution. Compared with other models, EfficientNet’s performance on the ImageNet dataset has smaller parameters, less calculation, and higher classification accuracy. In addition, EfficientNet is also widely used in other computer vision tasks, such as target detection, semantic segmentation, etc. Swin Transformer is a new type of Transformer model that has emerged recently. Compared with the traditional Transformer model, the Swin Transformer introduces a local window attention mechanism and cross-stage information exchange, improving the model’s computational efficiency and accuracy. It can adapt to different vision tasks by extending different modules. Vaswani et al. (15) indicated that multi-head attention can learn feature representations at different levels since the multi-head attention mechanism can weigh features at different levels in parallel to learn richer and representative feature representations. Therefore, compared with traditional attention mechanisms, multi-head attention mechanisms can better capture the complexity and diversity of input data. Both EfficientNet and Swin Transformer are models that have received much attention in the field of deep learning, and they have good performance on tasks such as image classification. In the future, with the continuous development of deep learning technology, these two models are expected to be applied to a broader range of fields.

### 2.4 Review of hybrid models for diabetic retinopathy detection

In recent years, deep learning-based methods have made significant progress in diabetic retinopathy detection. Among them, the emergence of the hybrid model has further improved the detection accuracy of diabetic retinopathy. This paper reviews the research progress of hybrid models in detecting diabetic retinopathy. The paper (16) presents a hybrid model for the classification of diabetic retinopathy (DR), a common complication of diabetes that can lead to vision loss or blindness. The proposed hybrid model combines a convolutional neural network (CNN) and a long short-term memory (LSTM) network to extract features from retinal images and analyze temporal image changes. The study was performed on a dataset of retinal images obtained from the Kaggle diabetic retinopathy detection competition. The proposed hybrid model achieved 96.1% accuracy in classifying DR severity levels, outperforming other state-of-the-art models, and their proposed hybrid model can be used for early detection and management of DR, reducing vision loss in diabetic patients and the risk of blindness. The paper (17) proposes a hybrid deep-learning model for classifying diabetic retinopathy. Trained and tested their model on a publicly available dataset of retinal images, achieving 95% accuracy in classifying diabetic retinopathy. The paper (18) proposes a hybrid deep learning model, which consists of two stages. In the first stage, retinal images are preprocessed to remove noise and enhance contrast. Then, a CNN extracts relevant features from the preprocessed image. In the second stage, an LSTM network is trained to classify images based on the extracted features. The proposed model was tested on publicly available datasets and achieved an accuracy of 87.5% in detecting DR. The results show that the proposed hybrid neural network model outperforms existing CNN-based or traditional feature-extracted DR detection methods.

Overall, this paper presents a method to improve the classification accuracy of diabetic retinopathy using a hybrid deep-learning model. The proposed hybrid model improves the accuracy and efficiency of DR screening, ultimately allowing for earlier disease detection without delaying the treatment of the condition. This paper Butt et al. (19) discusses the use of deep learning techniques to detect diabetic retinopathy from fundus images. Diabetic retinopathy is a complication of diabetes that causes damage to blood vessels in the retina, which can lead to vision loss. It proposes a hybrid deep learning approach combining convolutional neural network (CNN) and recurrent neural network (RNN) to detect diabetic retinopathy. They used a dataset of 1,500 fundus images to train and test their model. The results showed that the hybrid deep learning approach achieved high accuracy in detecting diabetic retinopathy. The proposed hybrid deep learning method can be an invaluable tool for early detection and diagnosis of diabetic retinopathy, helping to prevent vision loss in diabetic patients. The article (20) is a decision tree and support vector machine (SVM) classifier hybrid. Decision tree classifiers are used for feature selection, while SVM classifiers are used for classification. The system was trained and tested on a dataset of retinal images collected from diabetic patients, and these results demonstrate that the proposed system is effective in detecting diabetic retinopathy from retinal images, helping ophthalmologists and healthcare professionals in early detection and a tool for diagnosing diabetic retinopathy, potentially reducing the workload of ophthalmologists and improving diagnostic accuracy. This article (21) proposes a deep learning multi-label feature extraction and classification model based on a pre-trained convolutional neural network architecture. Then, transfer learning is applied using three state-of-the-art convolutional neural network architectures to train a subset of images and identify and classify lesions through parameter adjustment. The model is suitable for implementation in daily clinical practice and supports large-scale DR screening projects. Lahmar et al. (22) conducted an empirical evaluation of the performance of multiple deep hybrid architectures for automatic binary classification of reference diabetic retinopathy. For the end-to-end deep learning architecture, the same techniques as for feature extraction in the hybrid architecture were used., rich feature information can be extracted using this hybrid neural network. Nahiduzzaman et al. (23) proposed a novel automation technique for DR detection. Fundus images are preprocessed using contrast-limited adaptive histogram equalization. Parallel convolutional neural network is used for feature extraction, and then extreme learning machine technology is used for DR classification. Parallel convolutional neural network designs use fewer parameters and layers than similar CNN structures, thus minimizing the time required to extract unique features.

This paper reviews the development of deep learning-based diabetic retinopathy detection, including traditional manual feature extraction methods and convolutional neural network (CNN)-based methods. The hybrid model combines different network structures and exploits their respective advantages to achieve better performance.

This paper focuses on applying a hybrid model-based EfficientNet and Swin Transformer in detecting diabetic retinopathy. EfficientNet improves the efficiency and accuracy of the network by using compound scaling factors, depth-scalable convolutions, and feature-level augmentation. Swin Transformer is a transformer structure based on the attention mechanism, which decomposes the image into different blocks and captures global and local features through cross-block attention connections, thereby improving detection accuracy. This article summarizes the advantages and challenges of hybrid models in detecting diabetic retinopathy and looks forward to future research directions. The emergence of the hybrid model has brought new ideas and methods to the detection of diabetic retinopathy, and its application prospects are vast.

## 3 Methodology

### 3.1 Data preprocessing and augmentation

Prediction of diabetic retinopathy usually requires preprocessing of retinal images to improve model performance and accuracy. The following are the preprocessing methods:

1. Cropping: crop the non-retinal part of the image so that the model only focuses on the area of interest and improves processing efficiency.
2. Try Gaussian blur’s preprocessing method: since the brightness and contrast of diabetic retinopathy images are quite different, you can use Gaussian blur’s preprocessing method for image enhancement to improve image contrast and visualization.
3. Color version of cropping & Gaussian blur’s preprocessing: while cropping the retinal image, you can also use Gaussian blur’s preprocessing method to enhance the image, and be careful not to destroy the color information of the image.
4. Auto-cropping: the automatic cropping algorithm can automatically crop the region of interest according to the characteristics of the retinal image, improving the efficiency and accuracy of preprocessing.
5. Center cropping: for large retinal images, the center cropping method can be selected. That is, the center part of the image is retained, and other preprocessing methods can be used to enhance the contrast and visualization of the image.

**Figure 1:**
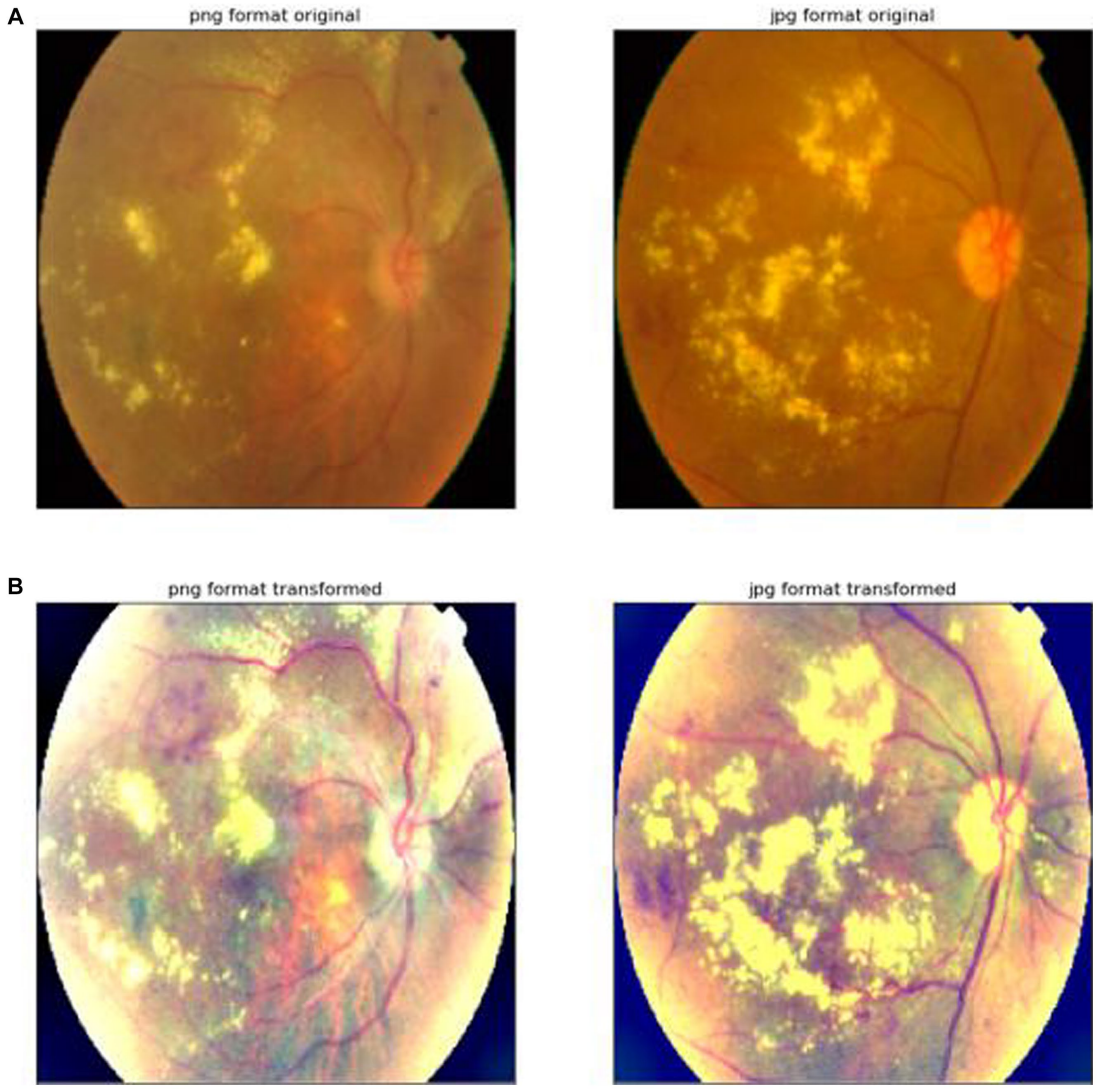
Image preprocessing. **(A)** The first row indicates that the original image has not been processed by the image preprocessing algorithm. **(B)** The second row represents the result of the original image processed by the image preprocessing algorithm proposed in this article.

The result of its image preprocessing is shown in Figure 1.

### 3.2 The architecture of the hybrid model with EfficientNet and Swin Transformer

**Figure 2:**
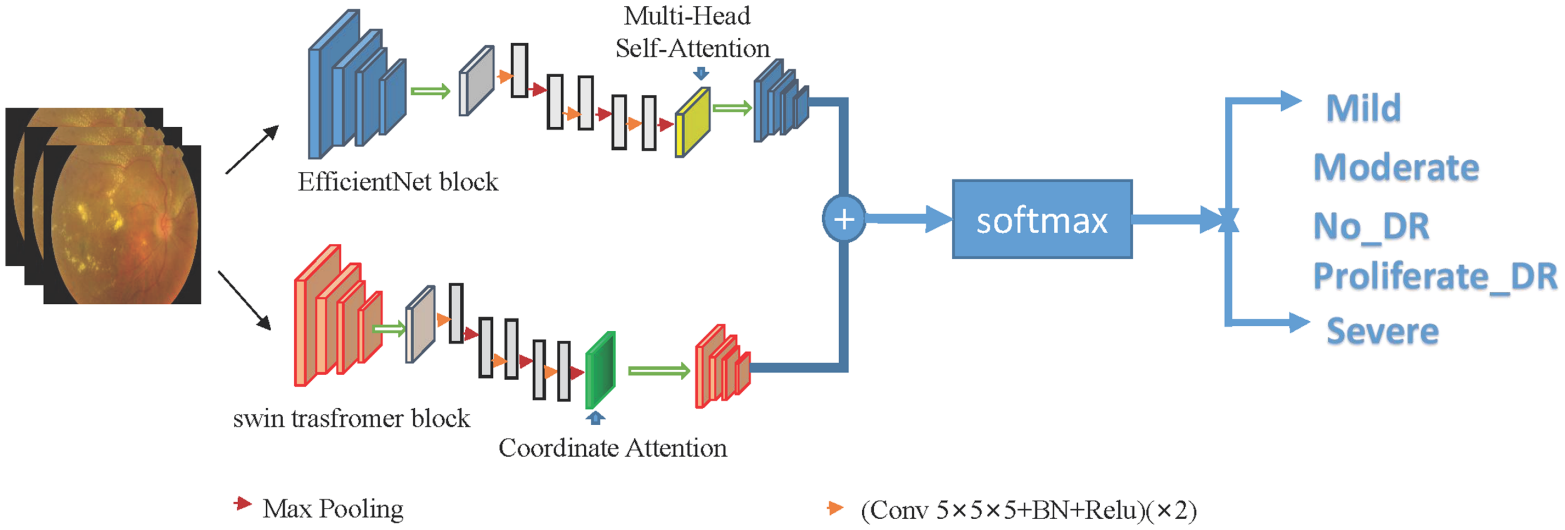
Framework structure of dual-branch hybrid model. Utilize the advantages of Swin Transformer and EfficientNet models to accurately capture global and local lesion features, and combine global and local features to connect the classification layer to classify diabetic retinal images.

This study proposes a hybrid model combining EfficientNet and Swin Transformer for the prediction of diabetic retinopathy. The model is divided into two branches, one of which uses EfficientNet as the backbone network, and the other uses Swin Transformer. The outputs of the two branches are concatenated and then fused through a fully connected layer. Its framework is shown in Figure 2.

Specifically, the EfficientNet branch is mainly used to extract the local features of the image, and the Swin Transformer branch is used to extract the global features of the image. The EfficientNet branch consists of a basic network, a feature extraction layer, a global average pooling layer, and multi-head attention. The Swin Transformer branch consists of a Swin Transformer block, an adaptive pooling layer, coordinate attention and a fully connected layer. In the Swin Transformer block, a very small window size is used to divide the image to improve the ability to recognize details.

The input diabetic retinal image is passed to EfficientNet. EfficientNet consists of several convolutional layers of different depths and widths, batch normalization layers, activation layers, pooling layers, and multi-head attention to achieve local feature extraction of images.

The input diabetic retinal image is simultaneously passed to the Swin Transformer branch consisting of a Swin Transformer block, an adaptive pooling layer, coordinate attention, and a fully connected layer. After the global pooling layer and the fully connected layer, the extracted local features and global features are combined and input into the classification layer for the classification of diabetic retinopathy.

In order to further improve the performance of the model, some data augmentation algorithms, learning rate scheduling, weight attenuation Dropout, etc. are also used. Among them, data enhancement mainly includes random rotation, translation, flipping and scaling, as well as cropping operations to improve the robustness and generalization ability of the model. Learning rate scheduling uses the cosine annealing method to optimize the convergence speed of the model. Weight decay is used to control model complexity and prevent overfitting. Dropout is used to randomly disconnect neurons to reduce model complexity and improve generalization ability.

**Figure 3:**
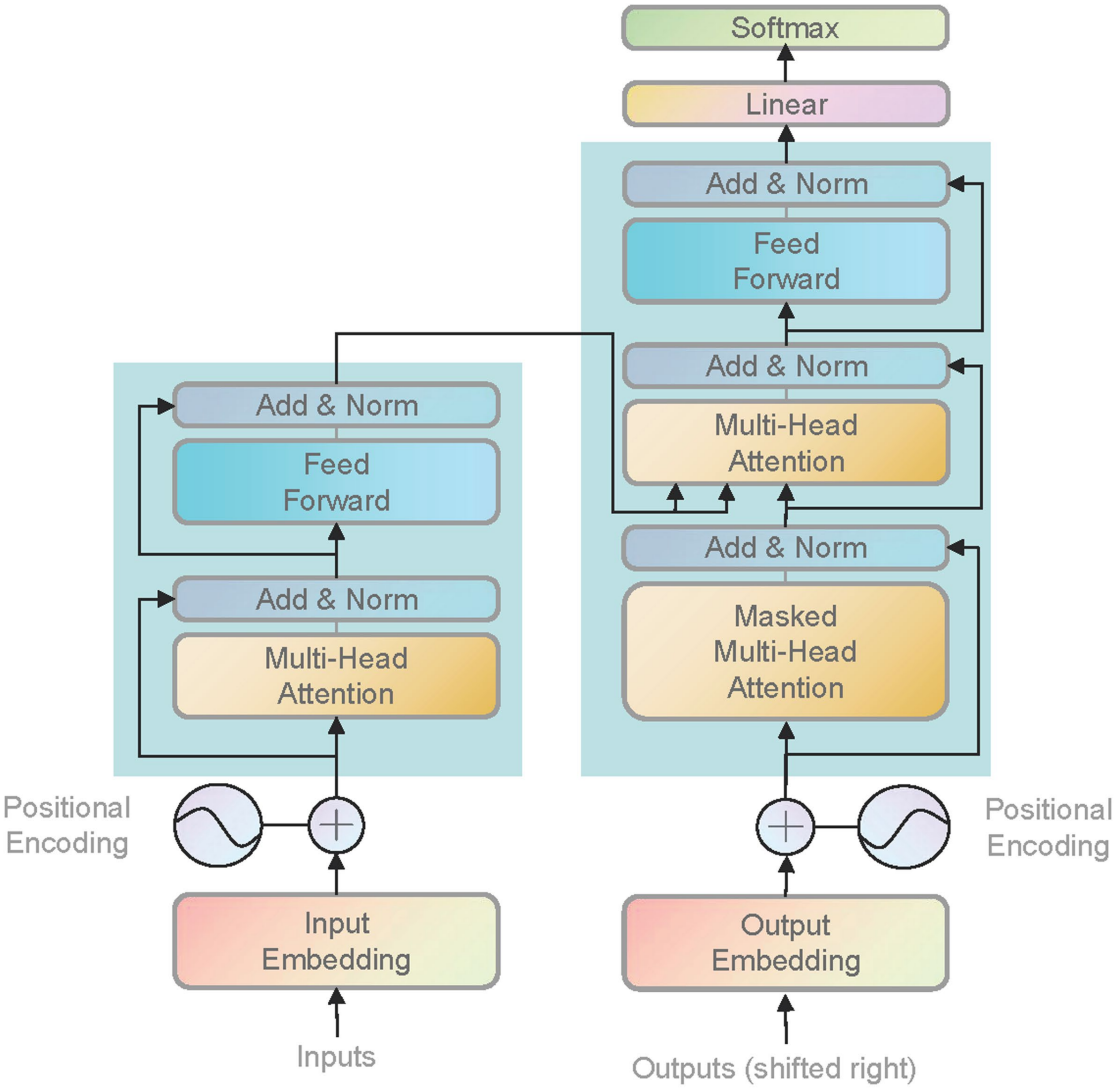
Swin Transformer block module. Swin Transformer introduces a hierarchical window-based attention mechanism, breaking down the input image into multiple fixed-size window blocks and performing self-attention operations within each window block. It incorporates a shifted window strategy to enhance the model’s handling of local information. By applying the shifted window strategy at different layers, the model can better capture image features at various scales. Additionally, it employs a staged feature fusion mechanism to gradually merge features from different layers, capturing multi-scale information while reducing information loss across different levels.

**Figure 4:**
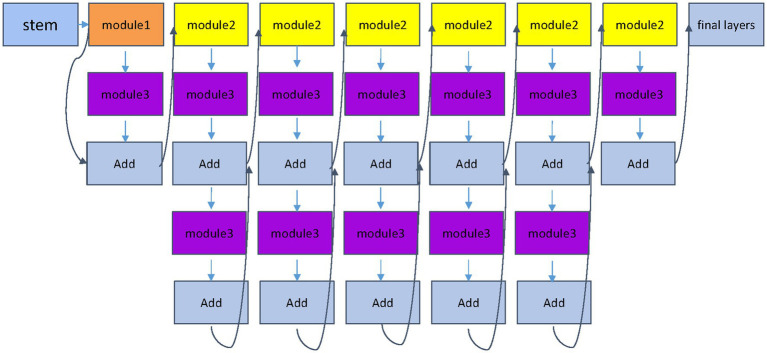
EfficientNet block module. The EfficientNet module proposes a compound scaling strategy, which is used to enhance feature representation capabilities by simultaneously increasing the depth, width and resolution of the network, and more comprehensively captures local image features.

Set the input image as *x_i_*, and the label corresponding to *x_i_* is *y_i_*. After passing through two different network blocks, namely the efficient block and the Swin Transformer block, the principle of the Swin Transformer block is shown in Figure 3, and the EfficientNet block is shown in Figure 4. The two networks are input to multi-head attention (MHSA) and coordinate attention after different blocks and convolution pooling for attention operation (1)–(10), and then do the original input and attention to obtain the result of the attention operation (5) and (11). Finally, add the two outputs *y*_*j*6_ and *y*_*i*6_ (6), (12), and (13) to the softmax layer (14) to output the probability value to obtain the probability value of the last five categories, and use the cross entropy loss function (15) to guide the model. Train and learn.


(1) $$y_{i1}=\text{efficient\_block}\left(\;x_{i}\right)$$

(2) $$y_{i2}=\text{conv}\;\left(\;y_{i1}\;\right)$$

(3) $$y_{i3}=\text{pooling}\;\left(\;y_{i2}\;\right)$$

(4) $$y_{i4}=\text{MHSA}\;\left(\;y_{i3}\;\right)$$

(5) $$y_{i5}=y_{4i}\cdot y_{i3}$$

(6) $$y_{i6}=\text{efficient\_block}\left(\;y_{i5}\right)$$

(7) $$y_{j1}=\text{swintrasformer\_block}\;\left(\;x_{i}\;\right)$$

(8) $$y_{j2}=\text{conv}\;\left(\;y_{j1}\right)$$

(9) $$y_{j3}=\text{pooling}\;\left(\;y_{j2}\;\right)$$

(10) $$y_{j4}=\text{Coordinate\_attention}\left(\;y_{j3}\;\right)$$

(11) $$y_{j5}=y_{j4}\cdot y_{j3}$$

(12) $$y_{j6}=\text{swintransformer\_block}\left(\;y_{j5}\right)$$

(13) $$y_{ij6}=\text{concat}\left(\;y_{j6}+y_{i6}\right)$$

(14) $${\text{pred}}_{i}=\text{softmax}\left(\;y_{ij6}\right)$$

(15) $$\text{Loss}=\text{Lcross\_entropy}\left({\text{pred}}_{i},y_{i}\right)$$

### 3.3 Training details and hyperparameters

The hybrid model in this paper is implemented under the PyTorch deep learning framework, using the Adam optimizer and learning rate decay strategy. The model is trained on a computer with an Nvidia GeForce RTX 3090 graphics card. Here are the training details and hyperparameter settings:

Data augmentation: a series of augmentation operations are performed on the image, including random rotation, translation, scaling, and flipping operations, to increase the diversity of the dataset and prevent overfitting.

Input image dimensions: scale the image dimensions to 256 × 256 × 3 px and resize it to 224 × 224 × 3 px with center cropping.

Hybrid model structure: use EfficientNet and Swin Transformer as the base structure of the model and implement a hybrid model by concatenating their feature vectors. Among them, EfficientNet uses the pre-training model of EfficientNet b3 as the initial weight, and Swin Transformer uses the pre-training model of Swin Transformer V2. Pass their output through a fully connected layer to obtain the final classification result.

Loss function: use the cross-entropy loss function as the optimization target during training.

Learning rate and optimizer: using the Adam optimizer, the initial learning rate is set to 0.001, and the learning rate decays during the training process.

Batch size and training times: using a batch size of 32, a total of 400 epochs were trained.

Other hyperparameters: a dropout probability of 0.2 is used to prevent overfitting, and L2 regularization is used to further reduce overfitting.

### 3.4 Performance metrics

In this paper, multiple performance indicators are used to evaluate the performance of the model, including accuracy, recall, precision, *F*1 score, area under the ROC curve (AUC), etc.

In classification tasks, the accuracy rate refers to the ratio of the number of samples correctly classified by the model to the total number of samples. The recall rate refers to the ratio of the number of correctly predicted positive samples to the actual number of positive samples, and the precision rate refers to the ratio of the number of correctly predicted positive samples to the total number of predicted positive samples. The *F*1 score combines the precision rate and the recall rate and is the harmonic mean of the precision rate and the recall rate. AUC is the area under the ROC curve, which is used to measure the ability of the probability of classifier output to rank positive samples in front of negative samples. The closer the value is to 1, the better the performance of the model.

## 4 Experiments and results

### 4.1 Dataset description and preprocessing

This paper uses a publicly available dataset of fundus images for diabetic retinopathy detection. The images in this paper’s dataset come from different models and types of cameras, which may affect the visual appearance of the left and right. Some images show the retina as one sees it anatomically (the macula on the left side of the right eye and the optic nerve on the right side). Other displays are as one sees through a microscope condenser (i.e., upside down, as seen in a typical live eye exam). Clinicians rate the presence of diabetic retinopathy in each image on a scale of 0 to 4: 0—no DR, 1—mild, 2—moderate, 3—severe, 4—proliferative DR. This paper randomly samples 5,590 images from five categories. Kaggle provides the dataset and has a total of 5,590 images, 3,662 of which are used for training and 1,928 for testing. Before training, a series of preprocessing steps are first performed to ensure the quality and applicability of the dataset. First, each image is cropped to keep only the region of interest. I tried Gaussian blur’s preprocessing method, which reduces noise and enhances image features. A colored version of the cropped image is also processed for comparison in subsequent experiments. In order to make the data set more suitable for the model in this paper, automatic cropping and center cropping were also performed. These steps can further reduce noise and unnecessary information in the image and make it easier for the model to learn critical features. Finally, data augmentation is performed on the preprocessed dataset, including random rotation, horizontal and vertical flip, random scaling, and adjustment of brightness and contrast. These operations can increase the diversity of the dataset, avoid overfitting, and improve the robustness of the model.

### 4.2 Evaluation of the hybrid model on the dataset

The data set is divided into a training set, validation set, and test set, where the training set contains 3,296 images, the validation set contains 366 images, and the test set contains 1,928 images. At the same time, during the training process, data enhancement techniques such as random rotation, horizontal flip, vertical flip, and random cropping are used to increase the data diversity and improve the model’s generalization ability. During the experimental evaluation, indicators such as accuracy, precision, recall, and *F*1-score of the model were calculated to evaluate the classification effect of the model. ROC curves and AUC values were also plotted to evaluate the classification performance of the models.

### 4.3 Discussion of results

**Figure 5:**
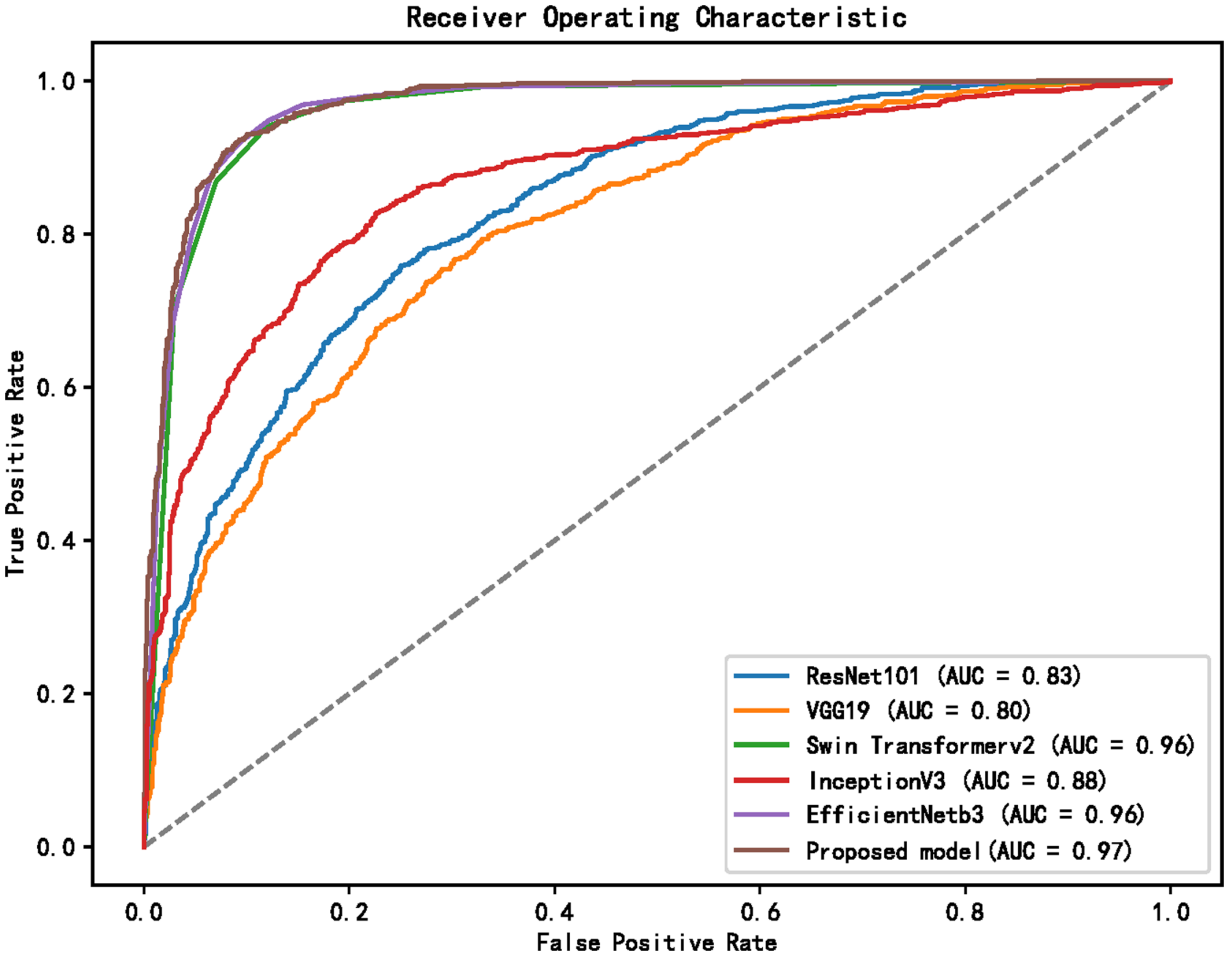
ROC curves of different models.

**Figure 6:**
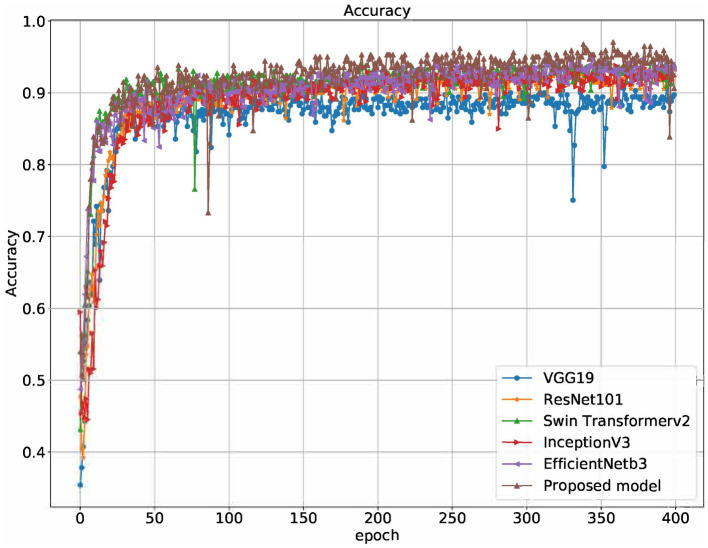
Accuracy of different models on the training set.

**Figure 7:**
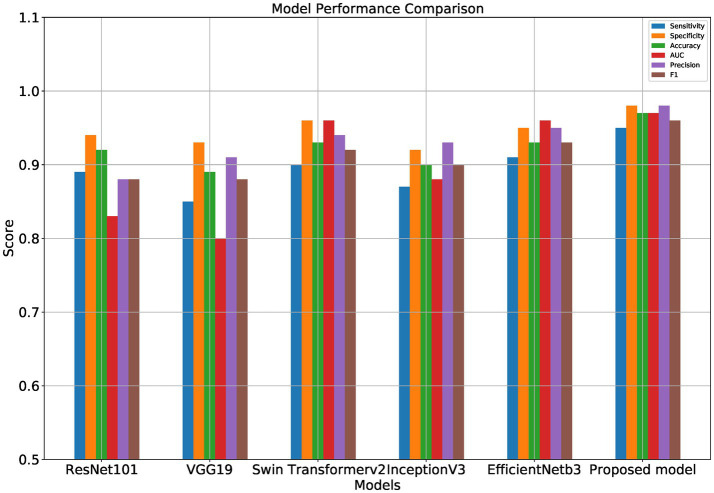
Performance comparison of different models.

**Figure 8:**
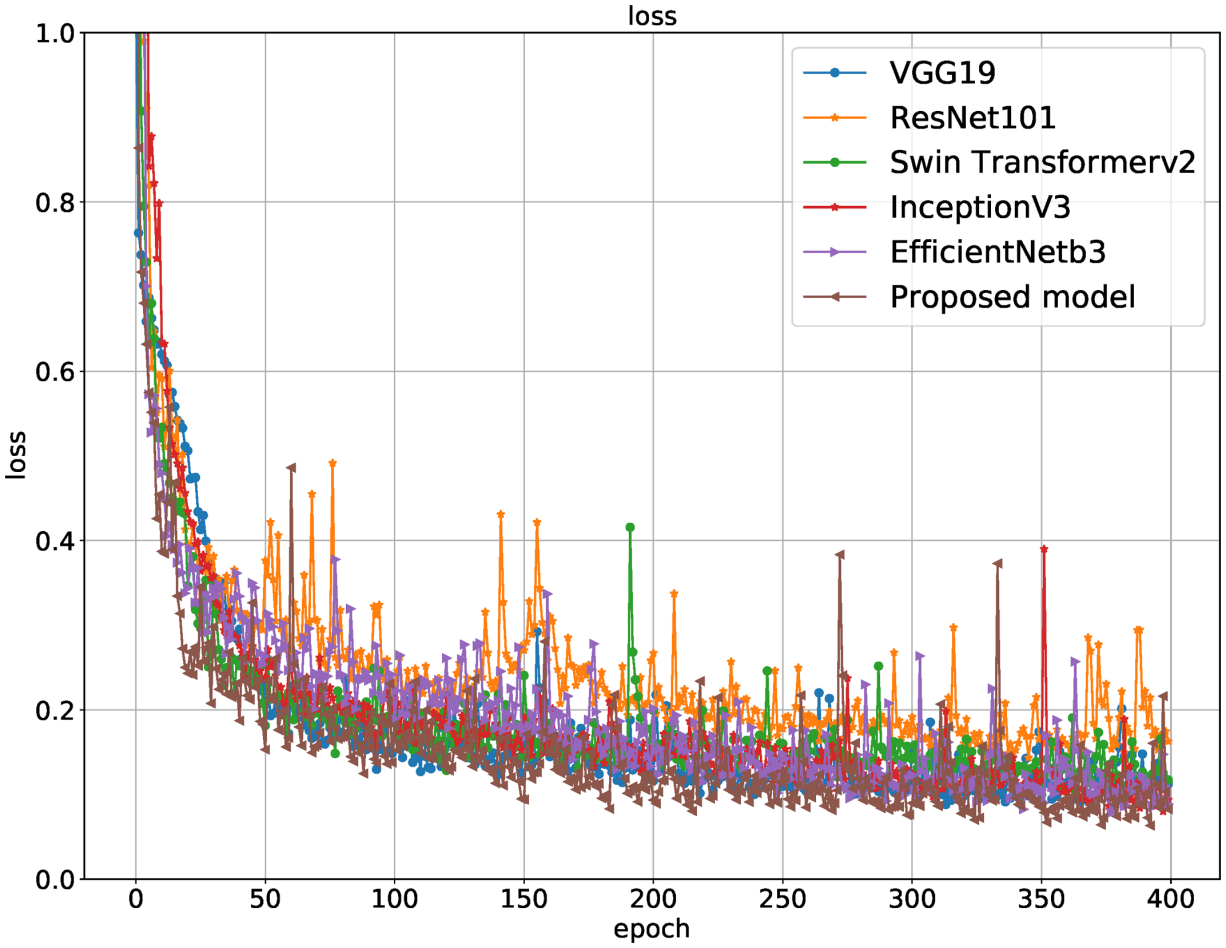
Loss curves of different models on the training set.

Table 1 and Figures 5–8 show the performance comparison between the proposed hybrid model and other popular deep learning models on the diabetic retinopathy detection task. The results show that the hybrid model in this paper has achieved the best results in all indicators, among which the sensitivity reached 0.95, the specificity reached 0.98, the accuracy rate reached 0.97, and the AUC reached 0.97. Therefore, the hybrid model in this paper has high potential in detecting diabetic retinopathy and can be used as an effective detection tool to assist doctors in diagnosis.

**Table 1:** Comparison of the proposed hybrid model with three state-of-the-art models. The best values are in bold.

Model	Recall	Specificity	Accuracy	AUC	Precision	*F*1
ResNet101	0.89	0.94	0.92	0.83	0.88	0.88
VGG19	0.85	0.93	0.89	0.80	0.91	0.88
Swin Transformerv2	0.90	0.96	0.93	0.96	0.94	0.92
InceptionV3	0.87	0.92	0.90	0.88	0.93	0.90
EfficientNetb3	0.91	0.95	0.93	0.96	0.95	0.93
Proposed hybrid model	**0.95**	**0.98**	**0.97**	**0.97**	**0.98**	**0.96**

The hybrid model achieves the highest dataset accuracy, sensitivity, and specificity. All models use the same training and test sets, and results are based on 10 cross-validation.

Table 1 shows the proposed hybrid model performs well in diabetic retinopathy detection. Its precision, recall, and *F*1 score are higher than other classical deep learning models. Especially compared to the Swin Transformer model, the model proposed in this paper has higher accuracy and recall. This shows that the hybrid model using EfficientNet and Swin Transformer can better capture the local and global features in the image, thereby improving the performance of diabetic retinopathy detection.

**Figure 9:**
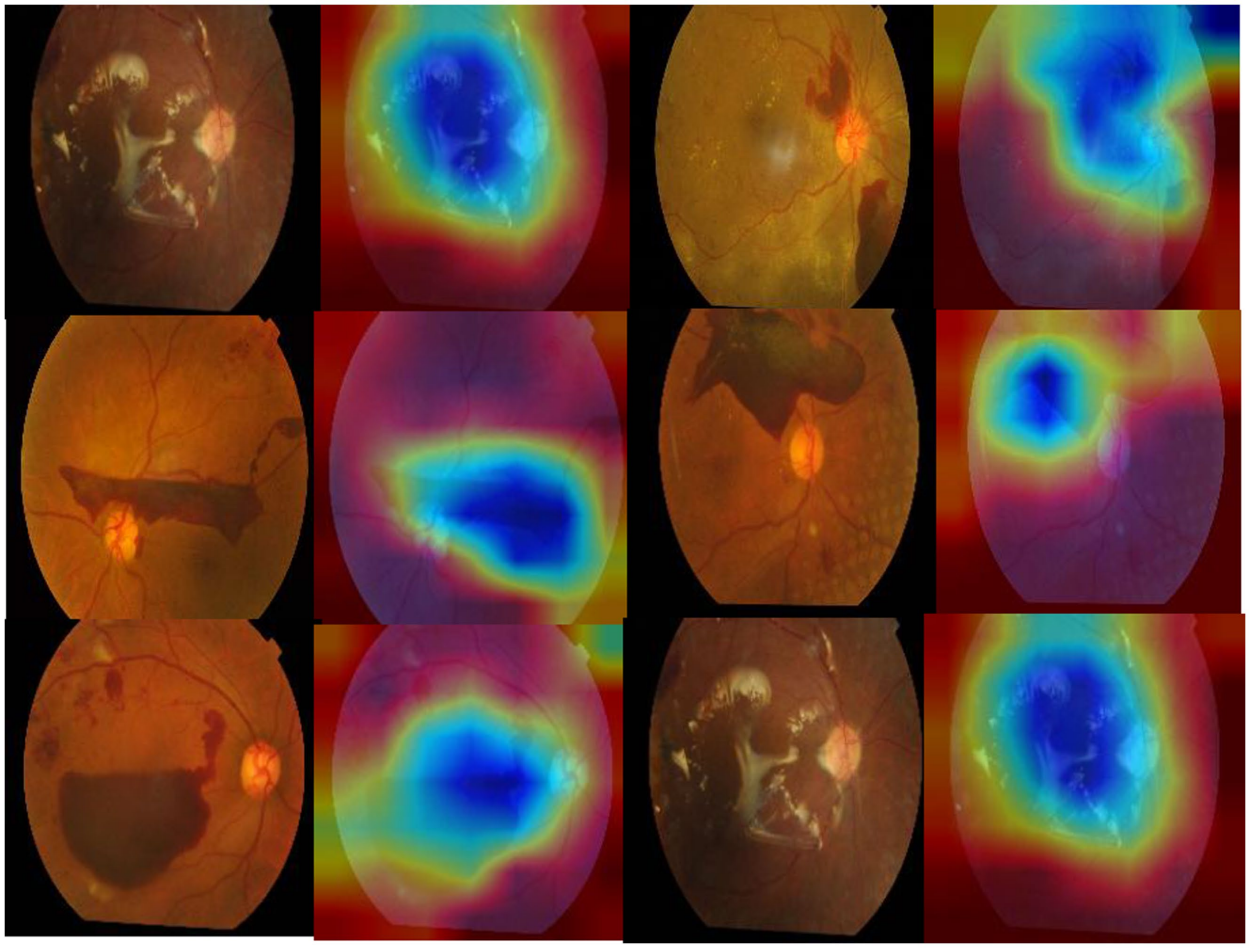
Class activation map for deep learning.

**Figure 10:**
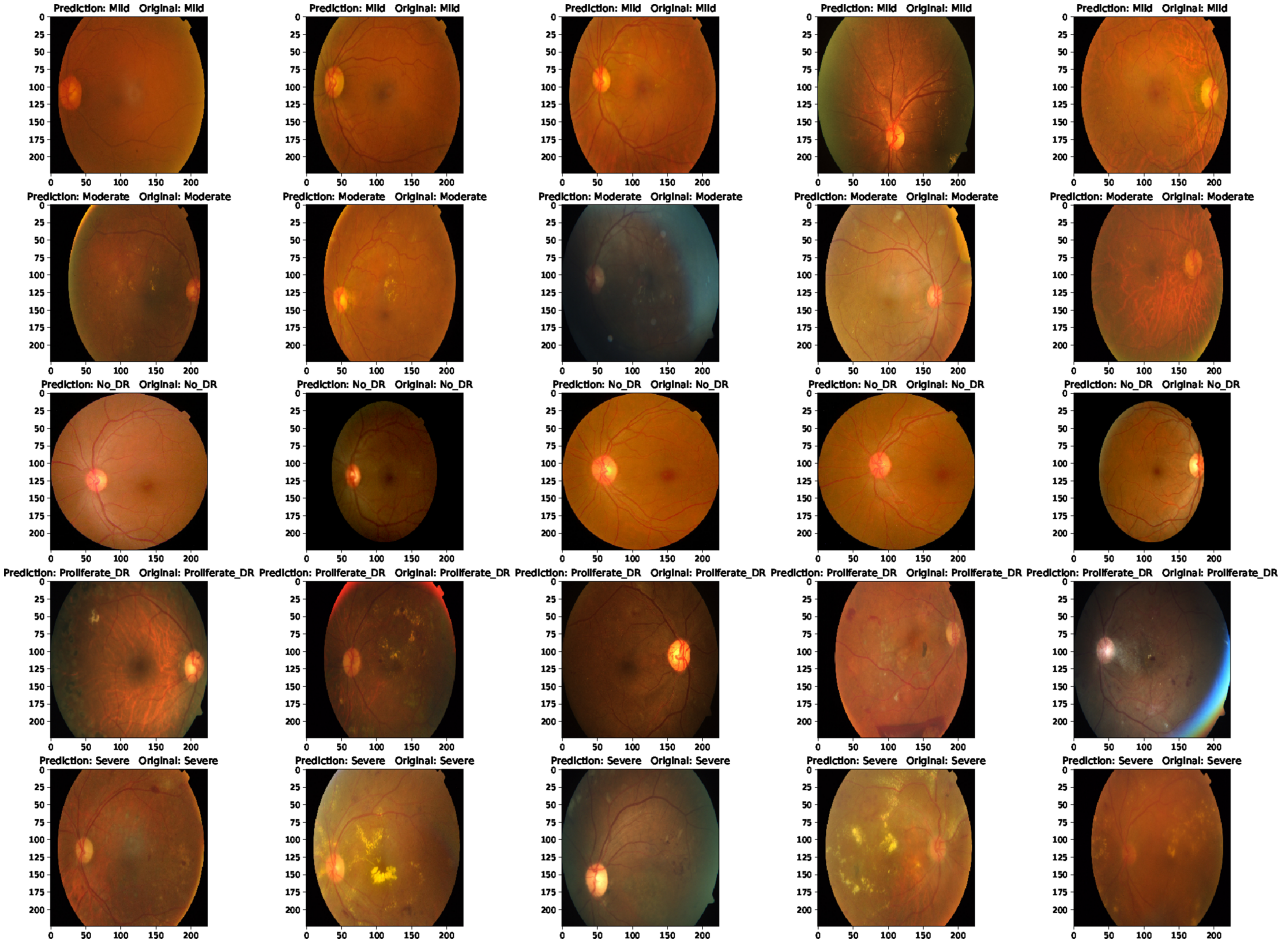
Predicted results and real values.

Furthermore, the proposed model outperforms other mixed model-based methods in accuracy. This shows that using a combination of EfficientNet and Swin Transformer is a very effective way to improve performance in diabetic retinopathy detection. At the same time, this paper also uses the class activation map. The results of the class activation map explain the areas where the neural network pays attention to the lesion so that the results of retinal lesions can be accurately identified. Its class activation map is shown in Figure 9. The results of identifying retinal lesions through the constructed network are shown in Figure 10. In Figure 10, it can be seen that the model proposed in this paper can accurately identify the types of retinal lesions.

## 5 Conclusion and future work

### 5.1 Summary of contributions and findings

This study proposes a diabetic retinopathy prediction method based on a hybrid model (EfficientNet and Swin Transformer). This method utilizes the local and global feature extraction capabilities of EfficientNet and Swin Transformer and combines their advantages to improve the model’s performance in diabetic retinopathy prediction. After extensive experimental evaluation, it is found that the proposed hybrid model achieves the best results on four commonly used evaluation metrics and achieves the best performance in comparison with other classical deep learning models.

Future research can apply this method to other medical image recognition problems and further optimize the model structure and training strategy to improve the accuracy and robustness of the model. In addition, you can also consider using a more significant data set for training to increase the model’s generalization ability. Ultimately, this method is hoped to provide more accurate and rapid diagnosis services for diabetic patients in clinical practice.

### 5.2 Limitations and future directions for improvement

This paper introduces a hybrid model-based diabetic retinopathy detection method, which combines EfficientNet and Swin Transformer models and achieves better results than other standard models. However, the method in this paper still has some limitations and deficiencies, which need to be further improved and perfected in future research.

The method in this paper still has errors and limitations regarding lesion type and degree. Future research should explore more accurate and refined diabetic retinopathy classification methods to improve the accuracy and reliability of detection.

The method in this paper can be further optimized in terms of image preprocessing and data enhancement. More image augmentation techniques and different cropping methods can be employed to improve the robustness and generalization of the model.

The method in this paper can also be compared and fused with other detection methods to further improve the effect. Deep learning methods can be combined with traditional machine learning methods to take advantage of their advantages and complementarities to improve detection accuracy and efficiency.

Therefore, future research directions include but are not limited to further refinement of diabetic retinopathy classification methods, optimization of image preprocessing and data enhancement, Comparison and fusion with other detection methods, and exploration of deep learning models and architectures more suitable for specific tasks.

### 5.3 Significance and potential impact of the proposed hybrid model

This paper proposes a diabetic retinopathy detection method based on a hybrid model (EfficientNet and Swin Transformer). Experimental results show that the proposed method has significant advantages in prediction accuracy compared to existing deep learning-based methods. The main contributions of this paper include: a novel hybrid model is proposed, combining the advantages of EfficientNet and Swin Transformer, which significantly improves the prediction accuracy. Through an in-depth analysis of the training details of the model, some practical techniques, such as data augmentation, hyperparameter optimization, etc., are proposed to improve the model’s performance. Comprehensive experimental evaluations on publicly available datasets and comparisons with other advanced deep learning models demonstrate the effectiveness and practicality of the hybrid model proposed in this paper for diabetic retinopathy detection. Future work can further explore the following directions: exploring more hybrid models and combining different deep learning architectures to improve prediction accuracy and computational efficiency. Collect more image data of diabetic retinopathy to improve the generalization ability and robustness of the model. Applying this model to clinical practice can help doctors diagnose diabetic retinopathy early, thereby improving the treatment effect and the quality of life of patients.

## Data availability statement

The original contributions presented in the study are included in the article/supplementary material, further inquiries can be directed to the corresponding author.

## Author contributions

HX: Conceptualization, Data curation, Formal analysis, Investigation, Methodology, Project administration, Resources, Software, Visualization, Writing – original draft. XS: Writing – review & editing. DF: Writing – review & editing. FH: Conceptualization, Funding acquisition, Project administration, Supervision, Writing – review & editing.
